# A Novel Controlled Release Immunosensor based on Benzimidazole Functionalized SiO_2_ and Cyclodextrin Functionalized Gold

**DOI:** 10.1038/srep19797

**Published:** 2016-01-21

**Authors:** Hongmin Ma, Yaoguang Wang, Dan Wu, Yong Zhang, Jian Gao, Xiang Ren, Bin Du, Qin Wei

**Affiliations:** 1 Key Laboratory of Chemical Sensing & Analysis in Universities of Shandong, School of Chemistry and Chemical Engineering, University of Jinan, Jinan 250022, China

## Abstract

A novel controlled release system-based sandwich-type immunosensor is fabricated to detect squamous cell carcinoma antigen (SCCA). The 1-methyl-1H-benzimidazole functionalized mesoporous SiO_2_ (MBI-MS) is used to load methylene blue (MB). β-cyclodextrin functionalized gold (CD-Au) is introduced as the gatekeeper for encapsulating MB and capturing the adamantly functional detection antibody (ADA-Ab_2_). And pH stimulus serves as the trigger system to control the MB release. After the load of MB, the CD-Au blocks the pores of the MBI-MS by the host-guest interaction in the neutral condition. However, when the pH is below 7.0, CD-Au is separated from the surface of MBI-MS owing to the protonation of the aromatic amines. The encapsulated MB is released from the pores of MBI-MS and detected by square wave voltammetry. The controlled release immunosensor shows a relatively wide linear range from 0.001 to 20 ng·mL^−1^ with a low detection limit of 0.25 pg·mL^−1^. The immunosensor also shows good reproducibility and selectivity, which endows it broad application prospect in clinical research.

Squamous cell carcinoma antigen (SCCA), as a member of the serine protease inhibitors family, is often used as a tumour marker with squamous cell carcinoma^1^,^2^. The SCCA normally exists in basal and parabasal layers of normal squamous epithelium with a low level, but it is found to be overexpressed in epithelia of cancerous tissue^3^. In the clinical diagnosis, an elevated level of SCCA has been identified as a prognostic factor in early-stage squamous cell carcinoma, and monitoring of SCCA levels after chemotherapy and operation provides important information about the recovery condition of patients^4^,^5^. Thus, the controlled release system-based sandwich-type immunoassay, which combines the specificity of immunoassay techniques and the high sensitivity of electrochemistry, is fabricated for the ultrasensitive detection of SCCA in this work.

In recent years, with the development of nanomedicine, mesoporous materials have been drawing attention from researchers owing to their uniform pore size, large surface area, good biocompatibility, low dielectric constant, low density, and low refractive index^6^,^7^,^8^. Mesoporous SiO_2_ (MS) is significant because it possesses characteristics of both silica and mesoporous materials. The versatility of silica chemistry provides a possibility to combine with other materials, such as noble metal, and fluorescent molecules^9^,^10^,^11^. At this point, the functionalized MS is widely used as nano-carriers in the drug transport and targeted drug delivery^12^,^13^. In this work, the 1-methyl-1H-benzimidazole functionalized MS (MBI-MS) is used as the carrier for loading more methylene blue (MB). The MBI-MS spheres with a radial diameter of ~50 nm contain ordered two-dimensional hexagonal arrays of tubular pores with diameters of ~2.8 nm. The nanopores are large enough to load MB, yet small enough to be closed by macrocyclic organic molecules, such as the β-cyclodextrin.

In addition, to control encapsulated cargo release, different trigger systems are introduced in the controlled delivery of encapsulated cargo, such as enzymes or redox, pH, light irradiation, magnetic, and electric stimuli^14^,^15^,^16^,^17^,^18^,^19^,^20^. Therefore, different trigger systems usually release their payload from nano-carriers as a response to corresponding stimuli. Our group has previously reported a novel controlled release system-based homogeneous immunoassay protocol based on specific interaction between antigen and antibody as the trigger systems and magnetic mesoporous Fe_3_O_4_ as a nanocontainer^21^. The fabricated homogeneous immunoassay protocol shows a relatively wide linear range and a low detection limit. Chen and coworkers reported the development and validation of the DNA labeling that leads to a unique amplification probe for the sensitive photoelectrochemical (PCE) immunoassay of HIV-1 p24 Antigen^22^. After the sandwich immunobinding, the DNA tags could be released by the H_2_SO_4_ stimuli, which induced PEC amplification and readout. Although some researches in the field of the biosensors have been reported, the sandwich-type electrochemical immunoassay methods based on controlled release system are still rare.

In this work, MBI-MS is used as the carrier for fabricating the controlled release system-based sandwich-type electrochemistry immunoassay. β-cyclodextrin functionalized gold (CD-Au) is introduced as the gatekeeper for encapsulating MB and capturing the adamantly functional detection antibody (ADA-Ab_2_). And pH stimulus serves as the trigger system for the controlled MB release. A graphical representation of the pH responsive MS nanovalve was shown in Fig. 1A. After the loading of MB, the CD-Au as a cap is connected to the MS through the host-guest interaction between cyclodextrin and 1-methyl-1H-benzimidazole (MBI) for blocking the nanopore openings and capturing the included MB molecules. However, acidic condition leads to protonation of the aromatic amines, followed by CD-Au cap release and MB diffusion from the nanopores. The released MB is detected by square wave voltammetry (SWV) and the achieved signal is proportional to the concentrations of SCCA. The proposed immunosensor shows a relatively wide linear range (from 0.001 to 20 ng·mL^−1^) and a low detection limit for the detection of SCCA (0.25 pg·mL^−1^).

## Experimental

### Regents and apparatus

SCCA, SCCA capture antibody (Ab_1_), and SCCA detection antibody (Ab_2_) were purchased from Beijing DINGGUO CHANGSHENG biotechnology CO. LTD (China). Adamantanecarboxylic acid (ADA-COOH) (99%) was obtained from J&K Chemical. Mercapto-β-cyclodextrin (HS-β-CD) was obtained from Shandong Zhiyuan Biotechnology Ltd. (China). Bovine serum albumin (BSA) was obtained from Sigma-Aldrich (Beijing, China). MBI was purchased from J&K Chemical (Beijing, China). Phosphate buffered solutions (PBS, 1/15 mol·L^−1^ KH_2_PO_4_ and 1/15 mol·L^−1^ Na_2_HPO_4_) were used as electrolyte for all electrochemistry measurements. All other chemical reagents were analytical reagents grade and directly used without further purification.

All electrochemical measurements were achieved on a RST 5200F electrochemical workstation (Zhengzhou Shiruisi Instrument Co. Ltd., China). Transmission electron microscope (TEM) images were recorded by a JEOL-1400 microscope (JEOL, Japan). Scanning electron microscope (SEM) images were obtained from JSM-6700F microscope (JEOL, Japan). FT-IR spectra were collected by using a FT-IR-410 infrared spectrometer (JASCO, Japan). Surface area measurements were performed on Micromeritics ASAP 2020M surface area and porosity analyzer (Quantachrome, United States).

### Preparation of MBI-functionalized MS

MS was prepared according to published literature procedures^23^ and used as the nanocontainer. In order to obtain MS-based nanocontainers, MBI was introduced on the nanopore entrances of MS in accordance with a previous report^13^. The MBI-functionalized mesoporous SiO_2_ (MBI-MS) was synthesized as follows: MBI (24 mg) was dissolved in 2.2 mL of N,N-dimethylformamide followed by the addition of tetrabutylammonium iodide (4 mg) and triethylamine (0.3 mL). Then 30 μL of chloromethyltrimethoxysilane was added into the above solution under magnetic stirring. The obtained solution was heated to 70 °C under N_2_ for 24 h. After the reaction was completed, the precipitates were separated by reduced pressure distillation and washed with hexane. The obtained product was dispersed in methylbenzene/ethanol suspension (*v:v* = 40:1) containing the as-prepared MS (200 mg). The mixture was heated to 115 °C under N_2_ for 12 h. The MBI-MS was separated by centrifugation (8000 rpm for 5 min), washed with methanol and dried at 50 °C in the oven.

### Synthesis of CD-Au

According to a reported process^24^, CD-Au was synthesized as follows: NaBH_4_ (75.5 mg) and HS-β-CD (8.0 mg) were dissolved into 20 mL of dimethylsulfoxide (DMSO) with stirring. This mixture was quickly added into another 20 mL of DMSO containing 0.24 mL of HAuCl_4_ (0.05 M). The mixture immediately turned dark brown, and then was continuously stirred for 24 h. Subsequently, the colloid was precipitated with 40 mL of CH_3_CN, which was obtained by centrifugation, washed with 60 mL of CH_3_CN:DMSO (*v:v* = 1:1) and 60 mL of ethanol, and dried under vacuum (60 °C) for 24 h.

### The functionalization of detection antibody

Adamantly functional detection antibody (ADA-Ab_2_) was synthesized as follows^25^: ADA-COOH (33 mg) was dispersed into 40 mL of ultrapure water with ultrasound. Then NaOH solution (1 M) was continuously dropped into the above suspension until the solid was dissolved completely. The obtained pellucid solution was immediately diluted to 100 mL (denoted as ADA-COOH solution). EDC (10 mg) and NHS (10 mg) were added into the mixture solution with 2 mL of ADA-COOH solution and 2 mL of PBS (50 mM, pH 7.4). After stirring for 30 min, 2 mL of Ab_2_ (100 μg·mL^−1^) was injected into the mixture solution, which was oscillated at 4 °C for 12 h. The prepared ADA-Ab_2_ was separated by centrifugation (8000 rpm for 10 min), washed with PBS (50 mM, pH = 7.4) and stored at −20 °C before use.

### Preparation of Ab_2_-CD-Au@SiO_2_ bioconjugates

The prepared MBI-MS (50 mg) was redispersed into MB solution (1 mM), which was stirred for 12 h. Then CD-Au (200 mg) was added into the suspension. After stirring for another 12 h, 400 μL of ADA-Ab_2_ (100 μg·mL^−1^) was injected into the above suspension and then stirred overnight. The suspension was separated by centrifugation (8000 rpm for 10 min) and the obtained solid (denoted as Ab_2_-CD-Au@SiO_2_) was stored at 4 °C before use.

### Fabrication of the immunosensor

**Figure 1:**
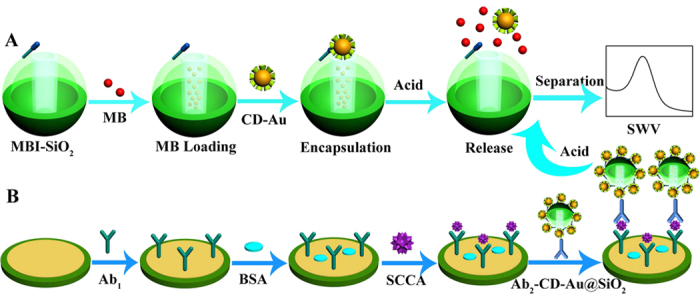
Graphical representation of the pH responsive MS nanovalve (**A**) and the fabrication process of the immunosensor (**B**).

Figure 1B displays the preparation process of the immunosensor. The gold electrode (AuE) with 4 mm diameter was polished with alumina powder of different particle sizes (1.0, 0.3, and 0.05 μm) and then activated in H_2_SO_4_ by cyclic voltammetry. Subsequently, 6 μL of Ab_1_ (10 μg·mL^−1^) was assembled onto the surface of the pretreated AuE with the interaction between Au and amidogen. After drying, 3 μL of BSA (1 wt%) was dropped to avoid non-specific adsorption and block possible remaining active sites. Different concentrations of SCCA were connected to sensor by specific immunity. Finally, 6 μL of prepared Ab_2_-CD-Au@SiO_2_ was modified and incubated at room temperature. After washing, the sensor was ready for measurement and stored at 4 °C before use.

## Results and Discussion

### Characterization of the MBI-MS

**Figure 2:**
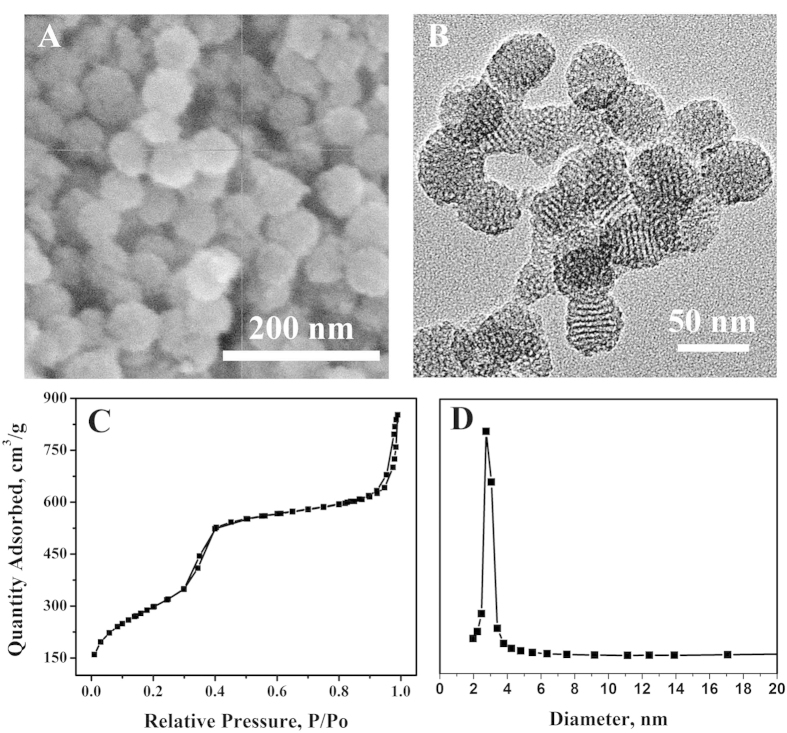
SEM image (**A**), TEM image (**B**), Nitrogen adsorption-desorption isotherm (**C**), and pore size distribution plots (**D**) of MBI-MS.

To confirm honeycomb-like structure of MBI-MS, SEM and TEM images are shown in Fig. 2A,B, respectively. SEM images of MBI-MS products show highly uniform spherical morphology with a radial diameter of ~50 nm. The ordered mesoporous structure of MBI-MS can be clearly observed on the surface of the nanosphere structure from the TEM image. In addition, the specific surface area and porosity analyzer were further characterized with the nitrogen sorption technique. The nitrogen adsorption-desorption isotherm and pore size distribution plots are showed in the Fig. 2C,D, respectively. It can be seen that the MBI-MS has a BET surface area of 1085 m^2^·g^−1^ and an average BJH pore size of 2.8 nm.

### Characterization of the CD-Au

**Figure 3:**
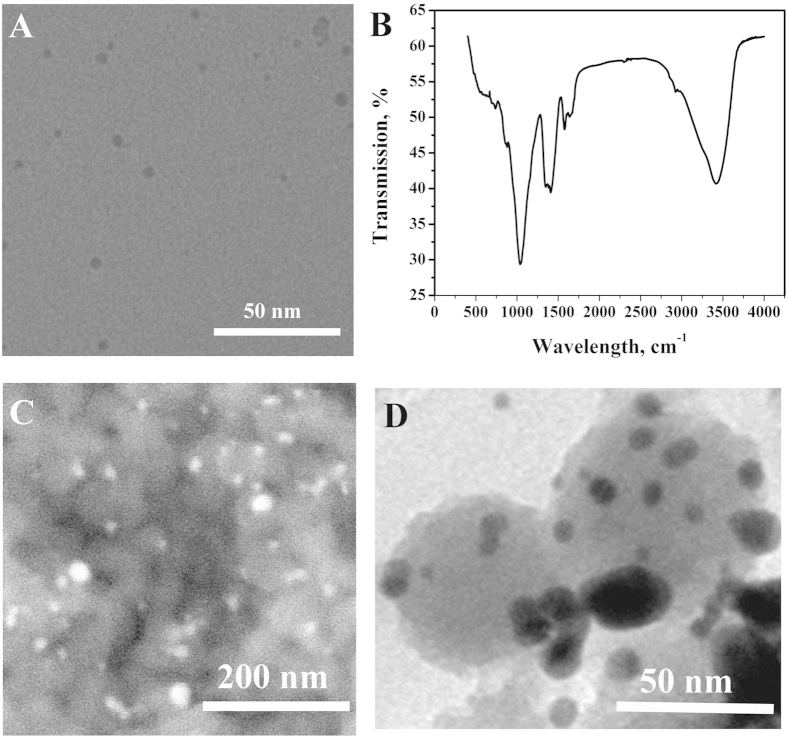
TEM image (**A**) and FT-IR spectra (**B**) of CD-Au, SEM image (**C**) and TEM image (**D**) of CD-Au@SiO_2_ compound.

The particle size and morphology of CD-Au were examined by TEM. As shown in the Fig. 3A, the CD-Au was found to have symmetric spherical structure with a diameter of ~4 nm. FT-IR spectrum of CD-Au was shown in the Fig. 3B. It was found that the characteristic absorption bands of β-CD at 3419 cm^−1^ (stretching vibration of O-H), 1405 cm^−1^ (the in-plane bending vibration of coupled O-H), 1038 cm^−1^ (the stretching vibration of C-O), 877 cm^−1^ (bending vibration of pyranose C-H), and 740 cm^−1^ (ring ‘breathing’ vibration), appeared at FT-IR spectrum of CD-Au. Therefore, it clearly confirmed that β-CD molecules were attached to the surface of Au.

### Characterization of the CD-Au@SiO_2_

In this work, SEM and TEM were used to investigate the feasibility of our design. Figure 3C showed the SEM image of CD-Au@SiO_2_ compound. It can be seen that many CD-Au nanoparticles were attached on the surface of MBI-MS, indicating that CD-Au@SiO_2_ compounds were prepared by host-guest interaction. The morphology of CD-Au@SiO_2_ compounds were further characterized through TEM (Fig. 3D). The CD-Au nanoparticles could be clearly observed on the surface of MBI-MS, which agreed with the SEM.

### Characterization of the immunosensor

**Figure 4:**
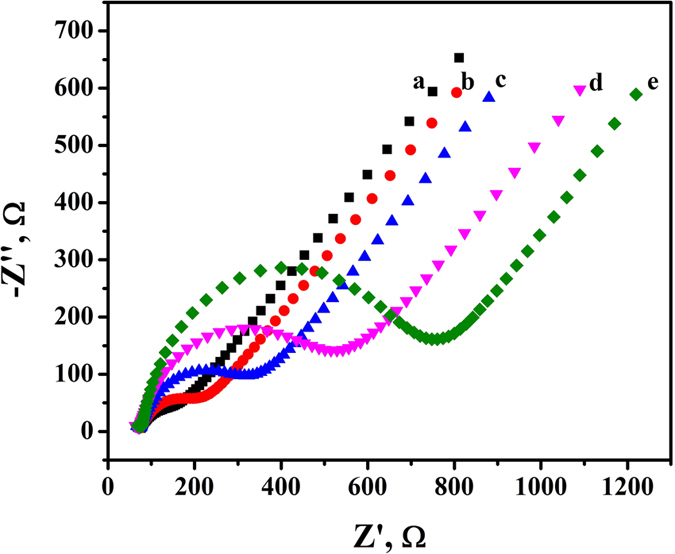
EIS obtained for different modified electrodes in Fe(CN)_6_^3-/4-^ solution (**a**) AuE, (**b**) Ab_1_/AuE, (**c**) BSA/Ab_1_/AuE, (**d**) SCCA/BSA/Ab_1_/AuE, (**e**) Ab_2_-CD-Au@SiO_2_/SCCA/BSA/Ab_1_/AuE.

The resistance value of the work electrode surface increases with the addition of cargo during the electrode modification process. Therefore, electrochemical impedance spectroscopy (EIS) was used to illustrate the fabrication of the immunosensor. As shown in Fig. 4, the bare AuE showed a minor semicircle diameter in high frequency region (curve a). After the modification of Ab_1_, the semicircle diameter increased (curve b) due to the fact that the protein film blocked the electron transfer. Similarly, semicircle diameter further increased with the addition of BSA (curve c), SCCA (curve d) and Ab_2_-CD-Au@SiO_2_ (curve e), implying that the biosensor had been fabricated successfully.

### Optimization of detection conditions

**Figure 5:**
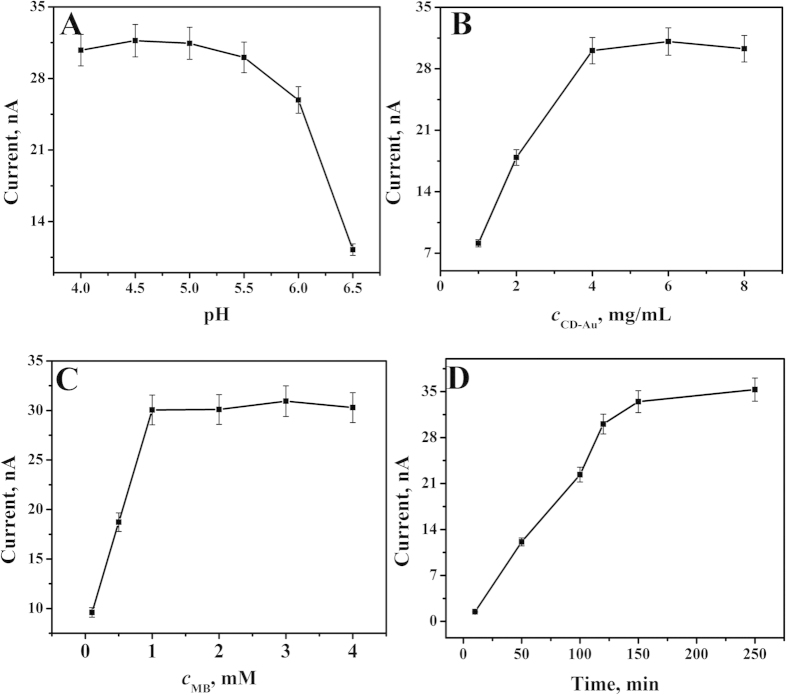
The optimization of experimental conditions with pH (**A**), CD-Au concentration (**B**), MB concentration (**C**) and release time (**D**).

The pH value of the detection solution has an important effect on the performance of the immunosensor. The acidic surroundings lead to protonation of the amines of MBI-SiO_2_, followed by CD-Au cap separation and the release of MB from the nanopores of MBI-SiO_2_, giving rise to the output of electrochemical singal of the immunosensor. Therefore, a series of pH values ranging from 4.0 to 6.5 were selected, and the current response began to flatten at pH < 5.5 in Fig. 5A. In addition, highly acidic surroundings would damage the activity of antibody and antigen, thus 5.5 was selected as the optimal pH.

The concentration of CD-Au is an important parameter affecting the number of encapsulated MB and the signal output. As shown in Fig. 5B, the signal increased with the increasing concentration of CD-Au from 1 to 4 mg·mL^−1^. With more than 4 mg·mL^−1^, the current response began to flatten, indicating that the number of connective CD-Au has reached basic saturated on the surface of MBI-SiO_2_. Thus, 4 mg·mL^−1^ of CD-Au was used in a follow-up experiment.

The concentration of MB has an important effect on the performance of the immunosensor. As shown in Fig. 5C, the current response of immunosensor increased sharply with the increasing concentration of MB and then began to flatten after 1 mM, indicating the number of encapsulated MB has reached maximum capacity of MBI-SiO_2_. Therefore, the 1 mM was chosen as the optimal concentration of MB.

The release time has an obvious effect on the feasibility of this method. As shown in Fig. 5D, the current response increased with the increase of release time. In order to obtain the optimal performance of the immunosensor and reduce the detection time, 120 min was used as the optimal release time in a follow-up experiment.

### Assay performance

**Figure 6:**
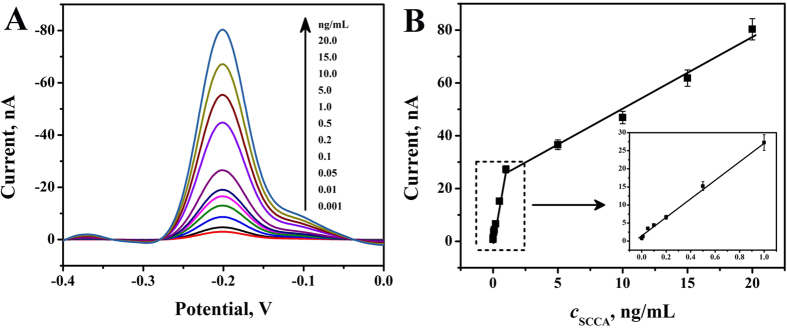
Typical SWV response curves of theimmunosensor for differentconcentration SCCA (**A**) and SWV peak currents versus various SCCA levels (**B**).

Under optimal conditions, the peak currents were proportional to the concentrations of SCCA in the range from 0.001 to 20 ng·mL^−1^. As seen in Fig. 6A, the well-defined SWV responses were observed and increased gradually with the increase of the SCCA concentration. In the Fig. 6B, the equation of the calibration plot was divided in two parts. Below 1.0 ng·mL^−1^, the equation was Y = 1.46 + 26.17X, r = 0.9982; over 1.0 ng·mL^−1^, the equation was Y = 22.56 + 2.74X, r = 0.9927. And the detection limit was estimated to be 0.25 pg·mL^−1^ (S/N = 3). The detection limit was lower than those in some previous reports^2^,^26^,^27^,^28^,^29^, such as 2.8 pg·mL^−1^, 0.02 ng·mL^−1^, 1.0 pg·mL^−1^, 8.53 pg·mL^−1^ and 0.17 ng·mL^−1^. The results indicated enough sensitivity for monitoring SCCA.

### Selectivity and reproducibility of immunosensor

To clarify the selectivity of the immunosensors, the current response of the immunosensor was researched toward interfering substances, such as carcino embryonie antigen (CEA), α-fetoprotein (AFP), prostate specific antigen (PSA), BSA and glucose. 1 ng·mL^−1^ of SCCA solution containing 50 ng·mL^−1^ of interfering substances was measured by the immunosensor and the measurements were shown in Figure S1A. The relative standard deviation (RSD) of current was less than 5% of that without interferences, which indicated the selectivity of the immunosensor was acceptable. In order to study the feasibility of the scheme, the reproducibility of the immunosensors was detected for 1.0 ng·mL^−1^ of SCCA. The results were shown in the Figure S1B that the RSD was 4.8%, suggesting that the precision and reproducibility of the proposed immunosensor were acceptable.

### Real sample analysis

To evaluate the feasibility and analytical reliability of the fabricated immunosensor, real samples were analyzed by using the standard addition method in human serum. After the addition of different concentrations of SCCA (0.5, and 1.0 ng·mL^−1^) into real samples and the following SWV detection, it can be seen from Table S1 that the average recoveries of the developed immunosensor were in the range of 96.7–107% and the RSD was 1.2–2.5%. In this sense, the good accuracy of the above immunosensor for clinical sample analysis can be proved accordingly.

## Conclusion

In this paper, a novel pH controlled release system-based sandwich-type immunosensor using CD-Au@SiO_2_ as a label is fabricated for SCCA. MB is encapsulated in the SiO_2_-based nano-carrier through host-guest interaction between CD-Au and MBI-SiO_2_. pH stimulus is used as the trigger system to control the CD-Au separation and the MB release. The current response produced by the electro-catalysis of the released MB is proportional to the concentrations of SCCA. The proposed immunosensor shows a wide linear range with a low detection limit and acceptable precision and selectivity. Moreover, the developed immunoassay displays excellent analytical performance for the detection of SCCA, indicating that it has broad application prospect in clinical diagnostics.

## Additional Information

**How to cite this article**: Ma, H. *et al*. A Novel Controlled Release Immunosensor based on Benzimidazole Functionalized SiO_2_ and Cyclodextrin Functionalized Gold. *Sci. Rep.* **6**, 19797; doi: 10.1038/srep19797 (2016).

## Supplementary Material

Supplementary Information
