# Supplementary material for: A Novel Controlled Release Immunosensor based on Benzimidazole Functionalized SiO2 and Cyclodextrin Functionalized Gold
Source: Sci Rep. 2016 Jan 21;6:19797. doi: 10.1038/srep19797 (PMC4726347; doi:10.1038/srep19797)
Supplement: Supplementary Information [file srep19797-s1.pdf]

Supporting Information

A Novel Controlled Release Immunosensor Based on Benzimidazole Functionalized SiO_2 and Cyclodextrin Functionalized Gold

Hongmin Ma, Yaoguang Wang, Dan Wu, Yong Zhang, Jian Gao, Xiang Ren, Bin Du, Qin Wei*

Key Laboratory of Chemical Sensing & Analysis in Universities of Shandong, School of Chemistry and Chemical Engineering, University of Jinan, Jinan 250022, China

Correspondence: sdjndxwq@163.com

Tel: (86) 531-82767872

Fax: (86) 531-82767367

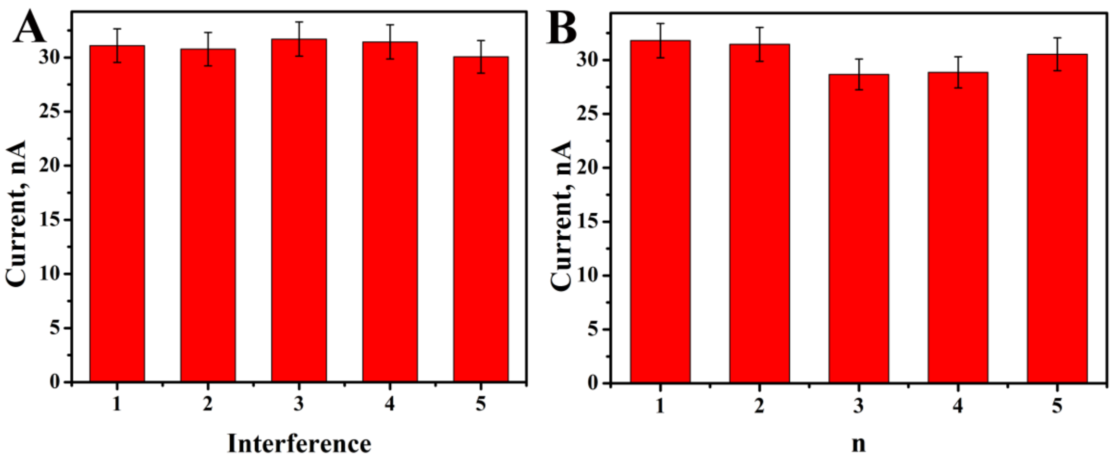

Figure S1. (A) Amperometric response of the immunosensor toward 1.0 ng mL^{-1} SCCA +50 ng mL^{-1} CEA (1), 1.0 ng mL^{-1} SCCA +50 ng mL^{-1} AFP (2), 1.0 ng mL^{-1} SCCA +50 ng mL^{-1} PSA (3), 1.0 ng mL^{-1} SCCA +50 ng mL^{-1} BSA (4) and 1.0 ng mL^{-1} SCCA +50 ng mL^{-1} glucose (5). (Error bar = RSD, *n* =5). (B) The reproducibility of the immunosensor.

Table S1 The results of the SCCA determination in serum sample.

Content of SCCA in the serum (ng mL^{-1})	The addition content (ng mL^{-1})	The detection content (ng mL^{-1}, *n*= 5)	RSD (%, *n*=5)	Recovery (%)
0.14	0.5	0.675，0.685，0.693，0.658，0.701	2.5	107
	1.0	1.093，1.094，1.126，1.102，1.101	1.2	96.7
